# Quantification of interplay and gradient effects for lung stereotactic ablative radiotherapy (SABR) treatments

**DOI:** 10.1120/jacmp.v17i1.5781

**Published:** 2016-01-08

**Authors:** Madelaine K. Tyler

**Affiliations:** ^1^ North Coast Cancer Institute Coffs Harbour New South Wales 2450 Australia; ^2^ Shoalhaven Cancer Care Centre Nowra New South Wales 2541 Australia

**Keywords:** 3DVH, SABR, interplay, 3D CRT, IMRT, VMAT

## Abstract

This study quantified the interplay and gradient effects on GTV dose coverage for 3D CRT, dMLC IMRT, and VMAT SABR treatments for target amplitudes of 5–30 mm using 3DVH v3.1 software incorporating 4D Respiratory MotionSim (4D RMS) module. For clinically relevant motion periods (5 s), the interplay effect was small, with deviations in the minimum dose covering the target volume (D99%) of less than ±2.5% for target amplitudes up to 30 mm. Increasing the period to 60 s resulted in interplay effects of up to ±15.0% on target D99% dose coverage. The gradient effect introduced by target motion resulted in deviations of up to ±3.5% in D99% target dose coverage. VMAT treatments showed the largest deviation in dose metrics, which was attributed to the long delivery times in comparison to dMLC IMRT. Retrospective patient analysis indicated minimal interplay and gradient effects for patients treated with dMLC IMRT at the NCCI.

PACS numbers: 87.55.km, 87.56.Fc

## I. INTRODUCTION

The dynamic nature of lung lesions in combination with the introduction of high dose perfraction radiotherapy treatments such as stereotactic ablative radiotherapy (SABR) introduces an uncertainty in target dosimetry due to low fractionation, inhomogeneous dose in the target volume, and target position within the treatment volume.^(1)^

Dynamic treatments such as intensity‐modulated radiation therapy (IMRT) and volumetric‐modulated arc therapy (VMAT) can create inhomogeneous dose distributions across the target volume. The inhomogeneity, combined with the dynamic nature of the target, creates uncertainty in whether the moving target (within the treatment volume) receives the dose as seen on the treatment planning system (TPS), which is planned on a static CT dataset.

Dosimetric effects of target motion can be separated into interplay and gradient effects. The dynamic nature of the target motion and the treatment delivery method (for IMRT and VMAT) contributes to the interplay effect, which can compromise target dose coverage.^(2)^ The interplay effect can be assessed in a single fraction by shifting the starting phase/position of the target and measuring the dose delivered to the target for a given dynamic delivery.^(3)^ Interplay does not affect 3D conformal radiotherapy (3D CRT) treatments due to the static nature of beam delivery.

The gradient effect is observed when the target moves out of a region of homogenous dose distribution. The gradient effect is generally negligible for conventional 3D CRT treatments where the target area is irradiated with a homogenous dose distribution. Exceptions can occur when tumor excursion occurs outside of the beam aperture or into the penumbral region of a field.

It has been reported that this effect increases as the amplitude of target motion increases.^(4)^ In SABR lung treatments, it is common practice to have an inhomogeneous distribution planned to the target volume, with large dose gradients and hotspots commonly accepted.^(1)^ In these situations where the dose distribution delivered to the target is deliberately inhomogeneous, gradient effects will have an impact on target dose coverage.^(3)^ As interplay is purely a temporal effect of target motion and delivery technique, in treatment deliveries where there is minimal or no interplay the gradient effect can be isolated.^(3)^

Numerous studies have been conducted for conventional fractionation for IMRT lung deliveries (~ up to 30 fractions) examining dosimetric errors induced by respiratory motion,^5^, ^6^, ^7^ concluding that errors induced by motion will average out for IMRT treatments over a conventional fractionation regime. Studies carried out on SABR and single‐fraction IMRT deliveries report dosimetric errors to the target of up to ±20%.^2^, ^5^, ^8^, ^9^

Effects of target motion on VMAT SABR treatments have been less extensively studied, with authors reporting that dosimetric effects on target coverage is minimal for small amplitudes of motion.^3^, ^10^, ^11^, ^12^ These studies (with the exception of Stambaugh et al.^(3)^) have used experimental methods (motion platforms and dynamic phantoms) to simulate motion induced by respiration, and report on the dosimetric differences between measured and planned distributions at a point (point dose), or in a plane (evaluated using gamma analysis). Whilst these studies provide an indication of the dose received by a moving target, none were able to estimate deviations in 3D dose coverage to the target volumes, which is of clinical significance in SABR where dose‐volume reporting is recommended.^13^, ^14^ Due to the nature of SABR treatment where hot‐spots are generally accepted in the treatment volume,^(1)^ the minimum (or near‐minimum) dose to the target volume is the main metric used for plan quality assessment^13^, ^14^ and is defined as the dose that covers 98% or 99% of the volume of interest (D98%, D99%).^(15)^

Stambaugh et al.^(3)^ used a prerelease version of the now commercially available 4D Respiratory MotionSim (4D RMS) module of 3DVH (Sun Nuclear Corporation, Melbourne, FL) software package to study the interplay and motion effects for ten retrospective VMAT patients. 4D RMS uses time‐resolved ArcCHECK (Sun Nuclear Corporation) measurements to create a 4D measurement‐guided dose reconstruction (4D MGDR). as described in detail by Nelms et al.^(16)^ This 4D MGDR is used to calculate dose to a moving voxel (or region of interest) by propagation of the voxel through the 4D dose reconstruction, and has been shown to accurately represent the motion‐perturbed dose for dynamic deliveries.^(17)^ A detailed summary of the validation and accuracy of the Respiratory 4D MotionSim can be found in the papers by Nelms et al.^(16)^ and Feygelman et al.^(17)^

The aim of this study was to critically assess intrafraction motion effects on target coverage for lung SABR (interplay effect), as well as the dose deviation between static and moving tumors (gradient effect) using 3DVH and 4D RMS for target motions of 5 mm – 30 mm. Simulations were performed of these target motion amplitudes using different breathing periods and treatment delivery techniques, using an anthropomorphic dynamic phantom (The Breathing Phantom (TBP), Model RS‐1500; Radiology Support Devices Inc., Carson, CA). Results from this study were used to provide clinical guidance and recommendations on the suitability of a particular treatment technique for patients undergoing SABR lung treatment at the North Coast Cancer Institute (NCCI), New South Wales, Australia.

## II. MATERIALS AND METHODS

SABR treatments were planned using Monaco v5.00.01 (Elekta, Stockholm, Sweden) on 4D CT scans of a dynamic breathing phantom (The Breathing Phantom RS‐1500) prior to ArcCHECK measurement, and analysis using 3DVH and 4D RMS.

The Breathing Phantom RS‐1500 is constructed to closely resemble a human thorax and simulate respiration. The phantom assembly consists of a lung balloon (filled with Styrofoam balls) inside a chest wall (containing bone and soft‐tissue materials). During respiration, the chest wall expands, replicating humanoid respiration effects on thorax anatomy. Inflation and deflation of the lung (simulating respiration) is controlled by a pump, with this volume of air programmed by the controller. Inside the right lung, a 2 cm diameter spherical target (tissue‐equivalent construction) is attached to a motor and control box, allowing motion of the tumor in the coronal plane of up to 30 mm (peak to peak). The volume of air in the lung, the breaths per minute, and the tumor amplitude are controlled via software attached to the phantom. In this study, amplitude of the target motion was set to 5, 10, 20, and 30 mm, with a 4D CT scan acquired for each target amplitude.

4D CT scans were acquired using a Siemens Somatom CT scanner (Siemens Medical Solutions, Erlangen, Germany) with ANZAI belt and load sensor apparatus (ANZAI Medical Co. LTD., Tokyo, Japan) to obtain the phantom breathing trace. The 4D CT scan consists of eight individual scan acquisitions each corresponding to phases of the breathing cycle and binned according to the percentage of the maximum amplitude of the breathing trace. An average intensity projection (AIP) scan is also reconstructed by the CT scanner and displays the average projection of the tissues from each of the individual phase scans and is used at the NCCI for SABR planning. All scans were imported into Focal 4D v4.80.00 (Elekta, Crawley, UK) for generation of a maximum‐intensity projection (MIP) CT dataset, which was subsequently used for delineation of the internal target volume (ITV). The GTV was contoured on each phase of the 4D CT dataset to enable location of the tumor center of mass on all phases for accurate motion simulation. The GTV in the 0% inspiration or full exhale phase was used as the motion region of interest (ROI) for dose calculation and simulation of motion in this study.

Monaco v5.00.01 treatment planning system (TPS) was used to generate SABR plans on the AIP CT scan for each target amplitude. For each amplitude, a VMAT, dMLC IMRT and 3D CRT plan was generated to deliver 48 Gy in 4 fractions to the target volume. Planning and organ delineation conformed to SBROCC 002^(18)^ and TROG 11.03 (SAFRON II)^(19)^ trial guidelines. A total of 12 plans (three for each target amplitude) were created and subsequently measured using the ArcCHECK on the linear accelerator. ArcCHECK results were used by 3DVH to create a 4D MGDR based on phantom measurements, as described by Nelms et al.^(16)^ The 4D MGDR for each SABR plan was subsequently imported into the 4D RMS module for simulation of motion and dose calculation to the target (GTV).

Anatomical motion of the target in the phantom was defined by using the center of the GTV contours (delineated from the 4D CT) from the 8 phases obtained from the 4D CT scan for each target amplitude. The target motion and the motion period are loaded in 4D RMS to calculate the dose delivered to the target during a treatment delivery. The breathing period was able to be altered manually in 4D RMS to simulate breathing periods of 5 s, 30 s, and 60 s to assess the temporal component of tumor motion on the SABR plans. Using this functionality, the target motion amplitude could be separated from the period of motion for analysis purposes. A summary of the simulations performed for each treatment technique is provided in Table 1.

**Table 1:** Target motion amplitudes and simulated motion periods for VMAT, dMLC IMRT, and 3D CRT SABR lung treatments simulated in this study.

*Target Motion Amplitude (mm)*	*Motion Period (s)*
5	5
	30
	60
10	5
	30
	60
20	5
	30
	60
30	5
	30
	60

Interplay and gradient effects were assessed by analysis of GTV, ITV, and PTV dose metrics including near‐maximum (D1% and D2%), mean (D50%), and near‐minimum (D98% and D99%) doses for each SABR plan with and without motion introduced. ICRU Report 83^(15)^ recommends the use of the D2% and D98% for reporting of IMRT treatments. Although these recommendations are relevant to SABR, the D1% and D99% metrics are presented to provide consistency with relevant previous publications and trial dose reporting guidelines.^3^, ^12^, ^13^, ^14^

### A. Interplay

To quantify the magnitude of the interplay effect, dose metrics for the GTV (D99%, D50%, D1%) were extracted from 4D RMS for a single fraction motion simulation of a treatment delivery with introduced shifts in the starting phase of the target motion relative to the beginning of the treatment beam. The magnitude of the phase shift was determined by dividing the total motion period (in seconds) for a simulation by the number of phases obtained from the 4D CT (eight). The phase shift introduced corresponded to 1/8, 2/8, etc., of the total motion period for a simulation. A total of eight simulations were performed for each target amplitude and motion period combination.

Dose metrics for the GTV (D99%, D50%. and D1%) were extracted for each simulation, with the maximum percentage deviation between any two starting phases calculated.

### B. Target motion (gradient) effects

GTV dose metrics (D99%, D50%, D1%) were extracted for each SABR treatment simulation for a static target (no motion introduced in 4D RMS) and with introduced target motion. The percentage deviation in dose metrics between the static and dynamic simulations was calculated.

Both a single‐fraction delivery and the total fractionation regime were simulated in 4D RMS to evaluate the effect of target dose coverage on small fractionation regimes typically encountered in SABR lung treatments.

In simulation of all fractions, 4D RMS introduces random phase shifts in the starting position of the target to match treatment, where the target is not likely to be in the same position for beam‐on for each fraction. The uncertainty introduced with random phase shifts in motion simulation was assessed by repeating a total fractionation simulation of the same plan ten times with the standard deviation (SD) between reported dose‐volume metrics calculated.

### C. Retrospective patient analysis

Four patients who had undergone dMLC IMRT SABR treatment at the NCCI were retrospectively analysed in 4D RMS to assess interplay effects and the effect of target motion on GTV dose coverage. Target motion was simulated by the position of the GTV at full expiration and full inspiration. As these patients were retrospectively examined, the CT scans (including ANZAI breathing traces) were not available from the CT scanner because they had been deleted following transfer to the planning system due to space constraints. As a result, a 5 s motion period corresponding to a normal respiration rate of 12 breaths per minute was used for all patients.

## III. RESULTS

### A. Interplay

**Figure 1:**
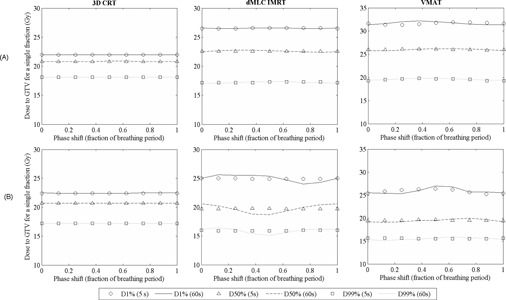
Effect of simulated phase shifts on a single‐fraction delivery on GTV dose coverage (D99%, D50%, D1%) for VMAT, dMLC IMRT, and 3D CRT SABR plans with (a) 5 mm and (b) 30 mm target motion amplitudes.

GTV dose metrics (D99%, D50%, and D1%) are plotted in Fig. 1 as a function of simulated phase shift for single‐fraction SABR treatments for 3D CRT, dMLC IMRT, and VMAT treatments with target motion amplitudes of 5 mm (smallest amplitude) and 30 mm (largest amplitude). To illustrate the effect of motion period on the interplay effect, both the 5 s (clinically relevant) and 60 s (not clinically relevant) motion periods are displayed.

**Figure 2:**
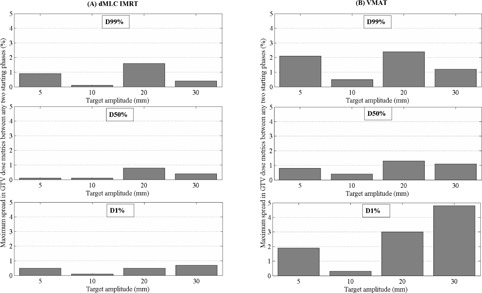
Maximum percentage deviation in D99%, D50%, and D1% dose metrics for the moving GTV between any two starting phases for a single fraction (a) dMLC IMRT and (b) VMAT SABR lung delivery for clinically relevant motion periods (5 s).

The largest percentage deviations in D99% and D50% GTV dose metrics across different starting phases was observed for the 60 s motion period for all target amplitudes. This trend was not observed for the near‐maximum dose (D1%). The larger target motion amplitudes had increased deviations in dose metrics, indicating greater interplay effects for these target motion amplitudes. Maximum percentage deviations in GTV D99%, D50%, and D1% from these simulations are plotted in Fig. 2 for a single‐fraction delivery simulation.

VMAT SABR delivery simulations showed larger interplay effects with deviations in D99%, D50%, and D1% of up to ±2.4%, ±1.3%, and ±4.8%, respectively, between any two starting phases for the 5 s motion period. Deviations increased as the motion period was increased to 60 s, with maximum deviations of ±5.2%, ±4.7%, and ±5.7% for the GTV D99%, D50%, and D1% coverage.

dMLC IMRT SABR deliveries showed minimal interplay effects for all target amplitudes for a clinically relevant motion period (5 s), with maximum percentage deviations between any two starting phases of ±1.6%, ±0.8%, and ±0.5%, respectively, for D99%, D50%, and D1% dose metrics. With the motion period increased to 60 s, the maximum deviation in dose metrics increased to ±14.0%, ±11.7%, and ±18.2%, respectively. The largest deviations were recorded for 20 and 30 mm motion amplitudes. For motion amplitudes of 10 mm and less, the interplay effect was minimal, with maximum deviations of ±1.5%, ±1.6%, and ±1.7% for D99%, D50%, and D1% coverage of the GTV.

As expected, interplay was negligible for 3D CRT deliveries, with maximum percentage deviation between two phases of motion of ±0.3%, ±0.4%, and ±0.0% for D99%, D50%, and D1% GTV dose metrics.

### B. Target motion effects

Table 2 shows the percentage deviation in the GTV D99%, D50%, and D1% dose metrics between a static and moving target, calculated by 4D RMS for target amplitudes of 5–30 mm, for single fraction and all fractions of the SABR delivery for a specific plan for the 5 s motion period. Motion periods of 30 and 60 s would not be encountered in a clinical situation and are therefore not displayed.

**Table 2:** Percentage deviation in GTV dose metrics between the static and moving target for SABR lung treatments with a 5s motion period. Deviations were calculated based on simulation of a single fraction delivery, and for the total number of fractions delivered for a treatment.

	*Target Motion Amplitude (mm)*	*Single Fraction*			*All Fractions*		
		*D99%*	*D50%*	*D1%*	*D99%*	*D50%*	*D1%*
VMAT	5	−0.5	−0.5	−0.4	0.0	−0.4	−0.1
	10	2.6	−1.7	−0.1	3.0	−1.2	−0.2
	20	−2.7	1.5	3.7	−3.4	1.4	4.9
	30	−2.1	1.3	7.8	−2.2	1.1	7.9
dMLC IMRT	5	−0.4	1.0	−0.3	−0.4	0.9	−0.2
	10	0.3	0.0	−0.1	0.4	0.0	−0.2
	20	−2.1	1.6	5.5	−2.7	1.9	5.4
	30	−2.9	2.0	5.3	−3.0	2.1	5.0
3D CRT	5	0.8	0.2	−0.2	0.7	0.2	−0.2
	10	0.2	−0.1	0.4	0.1	−0.1	−0.1
	20	1.1	0.4	−0.1	1.1	0.4	−0.1
	30	0.0	0.2	0.1	0.0	0.0	0.1

Differences in target dose coverage due to target motion (gradient effect) were assessed by comparison of DVH dose metrics for the same plan simulated with both a static and moving target. 3D CRT deviations in dose coverage were within ±1.1% for all target amplitudes. Dose coverage was found to differ by less than ±2.0% for dMLC IMRT deliveries with target amplitudes of 10 mm or less. For target amplitudes greater than this, deviations reached a maximum of ±5.5%. VMAT deliveries showed larger deviations of up to ±3.0% for the 10 mm amplitude, reaching a maximum of ±7.8% for the 30 mm target motion amplitude. The gradient effect was comparable between simulation of a single fraction delivery and delivery of the total fractionation (4 fractions) for the cases studied.

A single simulation was repeated 10 times in 4D RMS to assess the uncertainty in target motion simulation in the software. A maximum percentage difference of ±0.23% in dose metrics (D99%, D98%, D50%, D2%, D1%) for the GTV, ITV, and PTV was calculated, providing confidence in the accuracy of the motion simulation calculation done in 4D RMS.

### C. Retrospective patient analysis

Maximum interplay effects on GTV dose metrics (D1%, D50%, and D99%) averaged ±0.5% for all patients with a range of 0.1%–0.8%. The average percentage difference in target dose coverage for a moving target (gradient effect) for all patients was ±1.3%, ranging from 0.2%–2.3%.

## IV. DISCUSSION

The interplay effect is negligible, as expected, for 3D CRT treatments where the dynamic target moves within the static treatment aperture. In the case of dynamic deliveries (VMAT and IMRT) where the apertures (MLC, jaws) and the gantry (for VMAT treatments) are also moving, the interplay between the motion of the target and the treatment unit can affect the dose delivered to the target. Simulations performed using 4D RMS for dMLC IMRT SABR deliveries indicated that the interplay effect is minimal (<±2.4%) for D99%, D50%, and D1% dose metrics for the clinically relevant motion periods (5 s) for all target motion amplitudes. VMAT SABR deliveries showed an increased interplay effect with maximum deviation in the GTV D99%, D50%, and D1% dose metrics of up to ±4.8%.

The interplay effect increased as the motion period was extended to nonclinical periods of 30 s and 60 s, with maximum deviations in the GTV dose metrics of ±18.2% and ±5.7%, respectively, for dMLC IMRT and VMAT SABR treatments. Although motion periods of this length are not encountered clinically, the simulation of extended motion periods can be used to highlight the potential for interplay and its effect on target dose coverage. It is apparent that the interplay effect is larger for VMAT, as it is for dMLC IMRT, for the plans studied. This is attributed to long treatment times and slow gantry speeds encountered in VMAT, whereby the target motion period will have less of an effect on interplay. During this time there are significantly more breathing cycles, which hypothetically increases the time spent outside of the treatment aperture, resulting in an increased interplay effect for VMAT treatments. Planning studies conducted at the NCCI estimate an increase of 30%–40% in beam on time for VMAT compared with a nine‐field dMLC IMRT delivery for SABR treatments.

Increased modulation of VMAT plans compared to dMLC IMRT plans also contributed to the larger interplay and gradient effects, as observed in this study. The total MU delivered for each plan was comparable between the two dynamic techniques (within 2.5%). VMAT plans had a higher degree of modulation than dMLC IMRT plans, with lower MU per segment and an average of 187±40 segments for each plan. dMLC IMRT treatment plans averaged 122±4 segments. The average MU per segment was calculated as 20.5 and 32.4 for VMAT and dMLC IMRT plans respectively — equating to a 36% difference between techniques.

The gradient effect was determined to be nonnegligible for dynamic treatments (VMAT and IMRT) where inhomogeneous dose distributions are planned and the target encounters segments throughout the total treatment aperture for a beam/delivery. As a result, the target will often be close to, or outside, the treatment aperture, depending on the breathing cycle and the delivery.

This study has limitations in the representation of dose distributions relating to target motion in lung SABR patients. The phantom, although a good representation of a moving, human thorax complete with target motion, can only provide motion in the superior–inferior direction. Although the superior–inferior direction is the primary direction of motion for lung tumors,^(20)^ for the majority of patients, nonnegligible target motion also occurs in the anterior–posterior and medial–lateral directions. The amplitude of target motion is also highly dependent on the location of the lesion in the lung,^(21)^ which was not able to be investigated in this study. The results presented in this study focus on motion only in the superior–inferior plane and cannot necessarily be translated to motion effects in all planes without manipulation of the CT dataset (electron density and target contours) within the planning system.

Retrospective patient analysis showed interplay effects less than ±1.0% for all patients. The maximum deviation in dose coverage to the moving target compared to a static target (gradient effect) was less than ±2.5% for all patients. This indicates that for clinical cases, where target motion is small, there is minimal effect of target motion on the resulting dose distribution for lung targets. It must be noted that patient selection for dMLC IMRT SABR treatment is based on target size and amplitude at the NCCI. Patients who did not meet strict criteria were not treated using SABR. More retrospective patient analysis is needed to confirm initial results.

## V. CONCLUSIONS

The interplay effect was quantified for VMAT and dMLC IMRT SABR treatments. For clinically relevant motion periods (5 s), the interplay effect was small, with deviations in D99% dose metrics for the target volume of less than ±2.5% for target amplitudes up to 30 mm. Increasing the motion period up to 60 s resulted in interplay effects of up to ±15% on target dose coverage.

The gradient effect was examined for VMAT, dMLC IMRT, and 3D CRT by calculating differences in dose metrics for the GTV for the target when it is stationary, and when motion is introduced. Differences in dose coverage of the GTV greater than ±2% were recorded for target amplitudes of 20 mm and larger when motion was introduced for dMLC IMRT deliveries. VMAT treatments demonstrated a larger gradient effect, with deviations of up to ±3.0% for target motion of 10 mm. 3D CRT deliveries are largely unaffected by target motion as long as the target excursion remains within the treatment aperture. Minimal difference is observed between target dose coverage of simulations for single fraction SABR deliveries compared to simulations of the total treatment. This is due to the low fractionation (4 fractions) used for the SABR protocol chosen.

Retrospective patient analysis showed minimal effect of both interplay and gradient effects on GTV dose coverage for dMLC IMRT treatments.

## ACKNOWLEDGMENTS

The author would like to thank alphaXRT Limited (Auckland, New Zealand) and Sun Nuclear Corporation for providing a trial of the 3DVH and 4D RMS software for evaluation at the NCCI, and Mr Gareth Livingstone for his assistance in planning and input into scientific discussions relating to SABR.
